# MicroRNAs-1299, -126-3p and -30e-3p as Potential Diagnostic Biomarkers for Prediabetes

**DOI:** 10.3390/diagnostics11060949

**Published:** 2021-05-26

**Authors:** Cecil J. Weale, Don M. Matshazi, Saarah F. G. Davids, Shanel Raghubeer, Rajiv T. Erasmus, Andre P. Kengne, Glenda M. Davison, Tandi E. Matsha

**Affiliations:** 1 SAMRC/CPUT/ Cardiometabolic Health Research Unit, Department of Biomedical Sciences, Faculty of Health and Wellness Sciences, Cape Peninsula University of Technology, Cape Town 7530, South Africa; matshazid@gmail.com (D.M.M.); davidss@cput.ac.za (S.F.G.D.); raghubeers@cput.ac.za (S.R.); davisong@cput.ac.za (G.M.D.); 2 Division of Chemical Pathology, Faculty of Health Sciences, National Health Laboratory Service (NHLS) and University of Stellenbosch, Cape Town 8000, South Africa; rte@sun.ac.za; 3 Non-Communicable Diseases Research Unit, South African Medical Research Council, Cape Town 7505, South Africa; andre.kengne@mrc.ac.za; 4 Department of Medicine, University of Cape Town, Cape Town 7925, South Africa

**Keywords:** microRNA (miRNA), diabetes, prediabetes, Africa, biomarker

## Abstract

This cross-sectional study investigated the association of miR-1299, -126-3p and -30e-3p with and their diagnostic capability for dysglycaemia in 1273 (men, *n* = 345) South Africans, aged >20 years. Glycaemic status was assessed by oral glucose tolerance test (OGTT). Whole blood microRNA (miRNA) expressions were assessed using TaqMan-based reverse transcription quantitative-PCR (RT-qPCR). Receiver operating characteristic (ROC) curves assessed the ability of each miRNA to discriminate dysglycaemia, while multivariable logistic regression analyses linked expression with dysglycaemia. In all, 207 (16.2%) and 94 (7.4%) participants had prediabetes and type 2 diabetes mellitus (T2DM), respectively. All three miRNAs were significantly highly expressed in individuals with prediabetes compared to normotolerant patients, *p* < 0.001. miR-30e-3p and miR-126-3p were also significantly more expressed in T2DM versus normotolerant patients, *p* < 0.001. In multivariable logistic regressions, the three miRNAs were consistently and continuously associated with prediabetes, while only miR-126-3p was associated with T2DM. The ROC analysis indicated all three miRNAs had a significant overall predictive ability to diagnose prediabetes, diabetes and the combination of both (dysglycaemia), with the area under the receiver operating characteristic curve (AUC) being significantly higher for miR-126-3p in prediabetes. For prediabetes diagnosis, miR-126-3p (AUC = 0.760) outperformed HbA1c (AUC = 0.695), *p* = 0.042. These results suggest that miR-1299, -126-3p and -30e-3p are associated with prediabetes, and measuring miR-126-3p could potentially contribute to diabetes risk screening strategies.

## 1. Introduction

MicroRNAs (miRNAs) are endogenous, small (21–25 nucleotides in length), non-protein-coding but functional RNA [1] and their role in various metabolic disorders, including diabetes mellitus (DM), has drawn widespread interest. These miRNAs are present in tissues and several human body fluids such as peripheral blood at consistent and reproducible levels, and they are stable, as well as resistant to enzymatic digestion by RNAse [2]. It is for this reason that miRNAs have become potentially novel sources of biomarkers for diagnosis, prognosis and therapeutic options for a constellation of diseases [2,3].

Diabetes mellitus (DM), a condition affecting 463 million people worldwide [4], continues to pose a diagnostic dilemma in which for the last four decades plasma glucose and recently, HbA1c levels, remain the diagnostic and prognosis markers. Globally, nearly half of the subjects with diabetes remain undetected, contributing to the morbidity and mortality associated with DM. The hyperglycaemic state is a continuum of a prediabetes state, which is a term denoting impaired fasting glucose (IFG) and/or impaired glucose tolerance (IGT) [5]. Currently, an oral glucose tolerance test (OGTT) is required to identify individuals with IGT, a test that is considered to be invasive, cumbersome and lengthy. The identification of individuals with prediabetes is of importance since, at this stage, interventions such as diet or physical activity can prevent the progression to overt DM. Emerging evidence suggests that specific miRNAs could serve as potential biomarkers for prediabetes and DM. Among these is miR-126, in which a number of studies have shown its circulating levels to be decreased in subjects with type 2 diabetes mellitus (T2DM) [6,7,8]. We had earlier reported dysregulated miRNAs, including miR-126, in subjects with prediabetes or DM using high-throughput sequencing [9]. Herein, we selected the three most dysregulated miRNAs (hsa-miR-1299, hsa-miR-126-3p and hsa-miR-30e-3p) and investigated their association with and their diagnostic capability for dysglycaemia in a large independent sample, from an urban community residing in Cape Town, South Africa.

## 2. Materials and Methods

### 2.1. Study Design and Procedures

The cross-sectional data presented in this study were obtained from the ongoing Cape Town Vascular and Metabolic Health (VMH) study, described elsewhere [10]. The VMH is a population-based study which has been approved by the Cape Peninsula University of Technology (CPUT) Research Ethics Committee, as well as the Stellenbosch University Research Ethics Committee (respectively, NHREC: REC–230 408–014 and N14/01/003). The study enrolled mixed-ancestry participants who reside in Bellville South, located in the Northern Suburbs of Cape Town, Western Cape, South Africa. This population largely consists of mixed-ancestry individuals. According to South African census data from 2011, the population is comprised of 76.0% mixed ancestry, 18.5% black, 1.0% Asian, 0.5% Caucasian, and 4.0% individuals from other ethnicities [10,11]. The data collection for the current analysis took place between April 2014 and November 2016, involving only South Africans from Cape Town. Ethical approval for this sub-study was also sought from, and granted by the CPUT Research Ethics Committee (CPUT/HW–REC 2019/H3). A total of 1273 subjects including 207 prediabetes, 94 T2DM and 972 normotolerant individuals were enrolled in this study, and Informed Consent was obtained from the participants. All participants underwent the 75 g OGTT using the WHO criteria [12], blood pressure as well as anthropometric measurements. Prediabetes was defined as the presence of IFG and/or IGT [12].

At the time of screening, biochemical parameters were immediately analysed at an International Organization for Standardization (ISO) 15189 accredited Pathology practice (PathCare, Reference Laboratory, Cape Town, South Africa) as described elsewhere [9]. Blood glucose levels (mmol/L) were determined using an enzymatic hexokinase method (Beckman AU, Beckman Coulter, Cape Town, South Africa) and HbA1c levels were determined by High-Performance Liquid Chromatography (HPLC) (Biorad Variant Turbo, BioRad, Johannesburg, South Africa). Serum insulin was assessed by a paramagnetic particle chemiluminescence assay (Beckman DXI, Beckman Coulter, Cape Town, South Africa). High-density lipoprotein cholesterol (HDL-cholesterol) (mmol/L) was measured by enzymatic immuno-inhibition–End Point (Beckman AU, Beckman Coulter, Cape Town, South Africa), low-density lipoprotein cholesterol (LDL-cholesterol) (mmol/L) by enzymatic selective protection–End Point (Beckman AU, Beckman Coulter, Cape Town, South Africa) and serum triglycerides (Triglycerides-S) (mmol/L) were estimated by glycerol phosphate oxidase-peroxidase, End Point (Beckman AU, Beckman Coulter, Cape Town, South Africa). Ultra-sensitive *C*-reactive protein (usCRP) was measured using Latex Particle immunoturbidimetry (Beckman AU, Beckman Coulter, Cape Town, South Africa). γ-Glutamyltransferase (GGT) was assessed using International Federation of Clinical Chemistry and Laboratory Medicine (IFCC) standardised reagents on a Beckman AU (Beckman Coulter, Cape Town, South Africa). Serum cotinine was measured by Competitive Chemiluminescent (Immulite 2000, Siemens, Midrand, South Africa).

### 2.2. RNA Isolation

The total RNA, including miRNAs, was isolated from 3 mL of whole blood that had been collected in Tempus RNA tubes (Applied Biosystems, Life Technologies Corporation, Johannesburg, South Africa) that had been stored at −80 °C. The MagMAX for Stabilized Blood Tubes RNA Isolation Kit was used to perform the extraction, as per the manufacturer’s specifications (Ambion, Life Technologies Corporation, Johannesburg, South Africa). The purity and integrity of the subsequent RNA samples, each with a total volume of 50 μL, was then assessed using a Nanodrop (Nanodrop Technologies, Wilmington, DE, USA), and only samples with an RNA concentration >15 ng/mL, and an OD (optical density) ratio A260/A280 >1.8, were accepted for further processing.

### 2.3. Reverse Transcription Quantitative Real-Time PCR (RT-qPCR)

Following total RNA isolation, samples were converted to complementary DNA (cDNA) before further quantitative analysis. The subsequent reverse transcription was achieved using the TaqMan^TM^ Advanced cDNA Synthesis Kit, in accordance with the manufacturer’s specifications (Applied Biosystems, Thermo Fisher Scientific, Johannesburg, South Africa). This protocol involved producing cDNA using four separate reactions—poly(A) tailing, adapter ligation, reverse transcription, and the last step being an miR-Amp reaction. The aim of the poly(A) tailing reaction was to incorporate the addition of a 3’-adenosine tail to the miRNA present in the total RNA samples, catalysed by the enzyme poly(A) polymerase. A total of 2 μL of each total RNA sample was added to individual wells of a MicroAmp™ Optical 96-Well Reaction Plate. A reaction mix was then prepared with the poly(A) tailing reagents as per the manufacturer’s protocol, and 3 μL transferred into each well of the plate. Thereafter, the reaction plate was sealed with adhesion film, briefly mixed and centrifuged, and placed into a QuantStudio^™^ 7 Flex Real-Time PCR System for incubation (Applied Biosystems, Life Technologies Corporation, Johannesburg, South Africa). The QuantStudio^™^ 7 Flex was configured using the following settings and standard cycling—polyadenylation at 37 °C for 45 min; a stop reaction at 65 °C for 10 min; an infinite hold step at 4 °C. The plate was removed after incubation was completed, and proceeded to the adapter ligation step.

Following poly(A) tailing, the miRNA with the poly(A) tails underwent adaptor ligation at their 5′ end. The adaptors acted as the forward-primer binding sites for the miR-Amp reaction, which followed later. A reaction mix was prepared with the adapter ligation reagents, as per the manufacturer’s instructions, and 10 μL of the mix was transferred into each well of the reaction plate containing the poly(A) tailing reaction product. The plate was sealed, briefly mixed and centrifuged, and placed in the QuantStudio^™^ 7 Flex for incubation using the following settings—ligation at 16 °C for 60 min; an infinite hold step at 4 °C. Following this, the miRNA was reverse transcribed into cDNA. In this reaction, a universal RT primer bound to the 3′ poly(A) tails of the miRNA was used. Sufficient reaction mix was prepared as per the manufacturer’s guidelines, and 15 μL was added to each well of the 96-well reaction plate containing the adapter ligation reaction product. The plate was sealed, briefly mixed and centrifuged, and incubated in QuantStudio^™^ 7 Flex at the following settings—reverse transcription at 42 °C for 15 min; a stop reaction at 85 °C for 5 min; an infinite hold at 4 °C. The miR-Amp reaction step then followed. In this step, universal forward and reverse primers increased the number of cDNA templates present in each sample. A sufficient reaction mix was prepared in accordance with the manufacturer’s specifications, and 45 μL was transferred into a new 96-well reaction plate. A total of 5 μL of the reverse transcription reaction product from the previous step was added to each well of the new plate containing the miR-Amp reaction mix, and the plate was incubated in the QuantStudio^™^ 7 Flex set to the following cycling conditions—enzyme activation at 95 °C for 5 min, for 1 cycle; denaturation at 95 °C for 3 s, for 14 cycles; annealing/extension at 60 °C for 30 s, for 14 cycles; a stop reaction at 99 °C for 10 min, for 1 cycle; a hold step at 4 °C, for 1 cycle. Following successful completion of cDNA synthesis, samples were stored at −20 °C until required for quantitative PCR (qPCR) analysis.

In order to quantify the target miRNAs, pre-designed primers for each miRNA under investigation were used. These primers were miR-1299 (assay ID: 478696_mir; catalog number: A25576); miR-126-3p (assay ID: 477887_mir; catalog number: A25576); miR-30e-3p (assay ID: 478388_mir: catalog number: A25576) and they were executed in accordance with the TaqMan Advanced miRNA Assays and protocol on QuantStudio 7 Flex (Applied Biosystems, Thermo Fisher Scientific, Johannesburg, South Africa). Briefly, dilutions of 10^−1^ were made of each cDNA sample and 5 μL of each diluted cDNA sample was used for RT-qPCR. These were performed for optimum quantitative analysis. A sufficient reaction mix of the miRNA assay was prepared, according to the required number of reactions, and as per the manufacturer’s specifications. Samples and endogenous controls were all performed in duplicate. Data were obtained as cycle threshold (Ct) values, and the 2^−ΔCt^ method was used to assess the miRNA expression levels in each sample analysed, while the 2^−ΔΔCt^ value was used as the measure of the miRNA expression in each sample analysed compared with the control sample [13]. For analysis of miRNA expression levels, an endogenous control, miR-16-5p (assay ID: 477860_mir; catalog number: A25576), was used, of which pre-designed primers were obtained (Applied Biosystems, Johannesburg, Thermo Fisher Scientific, South Africa). The stability of the selected endogenous control was assessed and confirmed, and there was minimal variation between the glucose tolerance subgroups [14].

### 2.4. Statistical Analysis

Data analysis was performed using SPSS v.25 (IBM Corp, Armonk, NY, USA). Results were reported as count (and percentages), mean (and standard deviation) or median (25th–75th percentiles). The Chi-square test, analysis of the variance and Kruskal–Wallis test were used to compare baseline characteristics across glucose tolerance subgroups. The relationship between miRNAs and other variables was achieved by Spearman’s partial correlations adjusted for body mass index (BMI), age and gender. Multivariate logistic regression models were used to assess the association of miRNAs with prediabetes or DM with crude or adjusted odds ratio (OR). Models used were—Model 1: Crude; Model 2: which included age and sex; Model 3: which included age, sex and BMI; Model 4: included age, sex, BMI, systolic blood pressure (SBP), triglycerides, HDL- and LDL-cholesterol; and Model 5: which included age, sex, BMI, SBP, triglycerides, HbA1c, 2 h post-glucose, cotinine, HDL- and LDL-cholesterol; Model 6: included age, sex, waist circumference, 2 h post-glucose, fasting insulin, HDL-cholesterol and cotinine. The area under the receiver operating characteristic curve (AUC) was used to assess and compare the ability of each miRNA to predict the presence of prediabetes or DM. Then, the diagnostic ability of the miRNA with the highest AUC was assessed alongside HbA1c at their optimal sample-specific threshold, derived with the use of the Youden index methods. AUC comparison was based on the DeLong method, while the 95% confidence interval around the diagnostic performance measures was from bootstrap resampling, based on 2000 replications. A *p*-value < 0.05 was used to characterize statistically significant results.

## 3. Results

### 3.1. Basic Characteristics of the Study Subjects

Of a total of 1468 participants who consented, 184 with known type 2 diabetes and on treatment, as well as 11 with missing data, were excluded. The basic clinical characteristics of the remaining 1273 participants are summarised in Table 1. Over 70% of participants across all glucose tolerance statuses were women. Compared to the participants with normal glucose tolerance (NGT), those with prediabetes or diabetes were, on average, older, they had a higher waist circumference, BMI, blood pressure, triglycerides, ultra-sensitive *C*-reactive protein (us-CRP), LDL-cholesterol and GGT, all *p* < 0.001. Alcohol and tobacco consumption were significantly more prevalent in the NGT group, *p* ≤ 0.002.

### 3.2. Relative Expression of microRNAs (miRNAs)

**Figure 1:**
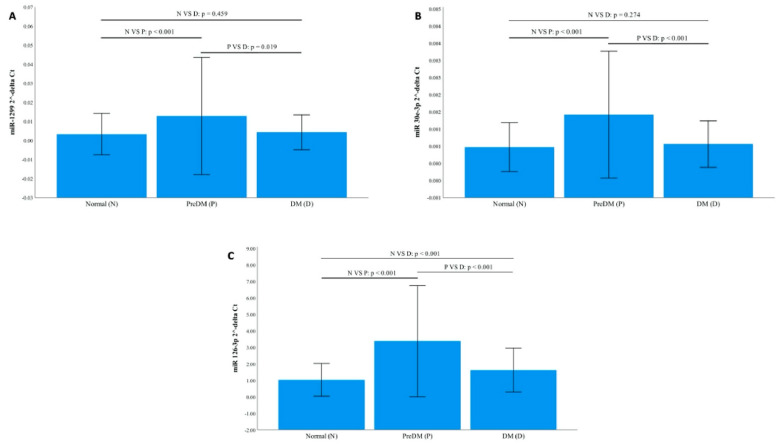
Relative Expression of miR-1299, miR-30e-3p and miR-126-3p according to glycaemic status. The expression of the microRNAs (miRNAs) was normalised to the relative expression of miR-16-5p. (**A**): miR-1299. (**B**): miR-30e-3p. (**C**): miR-126-3p. Data are shown as mean ± SD. *n*, normotolerant; D, diabetes; *p*, prediabetes.

All three miRNAs were significantly highly expressed in individuals with prediabetes compared to NGT, all *p* < 0.001. miR-30e-3p and miR-126-3p were significantly more expressed in T2DM compared to NGT, *p* < 0.001, while miR-1299 and miR-126-3p were significantly increased in prediabetes compared to T2DM, *p* ≤ 0.020 (Figure 1). These findings were further confirmed by the fold change analysis, 2^−ΔΔCt^. For instance, all three miRNAs were upregulated in prediabetes compared to the normal glucose tolerance group by a ≥3.12-fold change and downregulated in T2DM compared to prediabetes, ≤0.56-fold change (Table 2).

### 3.3. Correlation of miRNAs and Biochemical Parameters

We performed Spearman’s partial correlations and adjusted them for age, sex and BMI. The three miRNAs correlated positively which each other, *r* ≥ 0.743, *p* < 0.001, and the strongest correlation was observed between miR-30e-3p and miR-26-3p, *r* = 0.967, *p* < 0.001. All three miRNAs showed a negative correlation with waist circumference, r ≤ −0.398, *p* ≤ 0.040, but correlated positively with 2 h post-glucose and HDL-cholesterol, *r* ≥ 0.38, *p* ≤ 0.05 (Table 3).

### 3.4. Association between miRNAs and Prediabetes or Type 2 Diabetes

To investigate the association between the high expression of miR-1299, miR-30e-3p and miR-126-3p with prediabetes or T2DM, we performed multivariate logistic regression analysis. miR-1299 and miR-30e-3p expression level values (2^−ΔCt^) were too small, therefore, for interpretation purposes we converted the unit from 1 to 0.01 for these two miRNAs, meaning a 0.01 increase or decrease would represent a higher or lower odds of the condition. All three miRNAs were significantly associated with prediabetes when compared to individuals with NGT in crude and adjusted models for age, sex, BMI or waist circumference, SBP, HbA1c, triglycerides, HDL- and LDL-cholesterol, HbA1c, 2 h post-glucose, serum cotinine, odds ratio (OR) ≥ 1.26, 95% confidence interval (CI): ≥ 1.07–1.28, *p* ≤ 0.007). For T2DM, only miR-126-3p showed an association when compared to NGT, OR ≥ 1.43 (1.21–1.69, *p* < 0.001), but only after adjustment of age, sex, BMI, SBP, triglycerides, HDL- and LDL-cholesterol (Table 4). When we assessed the association between the miRNAs and T2DM using prediabetic individuals as the reference in multivariate logistic regression models, all three miRNAs were associated with a reduced risk of developing T2DM, OR, ≤0.76 (0.59–0.99, *p* ≤ 0.042). However, when the models were adjusted, only miR-30e-3p and miR-126-3p remained significant, OR, ≤0.73 (0.62–0.87, *p* ≤ 0.001) (Table 5).

### 3.5. Diagnostic Specificity and Sensitivity of the miRNAs for Prediabetes and Type 2 Diabetes

**Figure 2:**
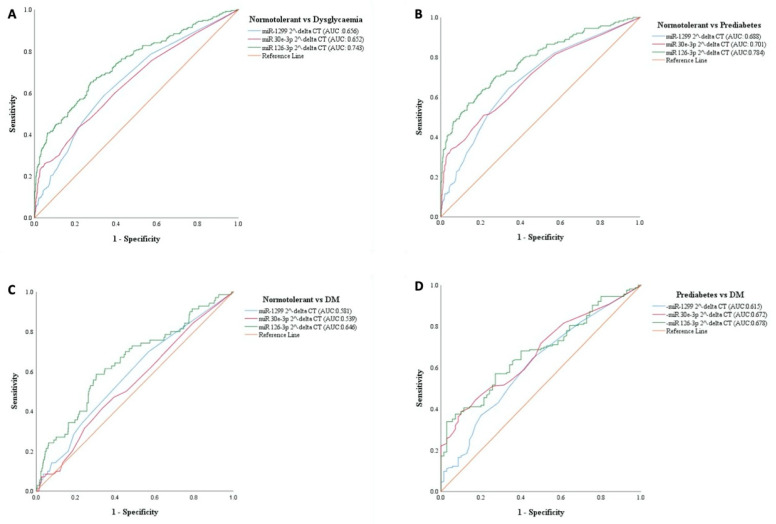
Receiver operating characteristic (ROC). ROCs were constructed for each miRNA to evaluate their diagnostic value for prediabetes, diabetes, dysglycaemia as positive cases and normotolerant people as negative cases, as well as for diabetes as positive cases and prediabetes as negative cases. (**A**): Dysglycaemia vs. normotolerant (**B**): Prediabetes vs. normotolerant (**C**): T2DM vs. normotolerant (**D**): Prediabetes vs T2DM.

Receiver operating characteristic (ROC) curves were drawn to investigate the diagnostic accuracy of the miRNAs as surrogate biomarkers for prediabetes and/or T2DM versus the normotolerant individuals. Furthermore, we investigated whether the miRNAs could serve as surrogate markers between prediabetes and T2DM. As shown in Figure 2, the best overall diagnostic accuracy was achieved for prediabetes versus NGT, particularly using miR-126-3p, area under the receiver operating characteristic curve (AUC) 0.76 (95% confidence interval [CI], 0.72–0.80, *p* < 0.0001) (Figure 2). All three miRNAs were of less value with regards to T2DM compared to NGT (Figure 2), while showing some value in differentiating between prediabetes and T2DM versus normotolerant people with miR-126-3p being the most sensitive, 0.67 (0.61–0.73, *p* < 0.0001) (Figure 2).

Compared with HbA1c, miR-126-3p had the same overall diagnostic accuracy for dysglycaemia (the combined outcome of prediabetes and T2DM) with AUCs of 0.718 for miR-126-3p vs. 0.753 for HbA1c (*p* = 0.214 for AUC comparison). For prediabetes diagnosis, miR-126-3p (AUC = 0.760) outperformed HbA1c (AUC = 0.695), *p* = 0.042; while for diabetes diagnosis, HbA1c (AUC = 0.861) largely outperformed miR-126-3p (AUC = 0.574), *p* < 0.0001. Measures of the diagnostic performance of both markers in diagnosing dysglycaemia, prediabetes and diabetes at their respective sample-specific optimal thresholds are shown in Table 6. These performance measures were always better for miR-126-3p in comparison to HbA1c for the outcome of prediabetes, mostly overlapping across the two markers for dysglycaemia diagnosis, and largely better for HbA1c vs. miR-126-3p for the outcome of T2DM.

## 4. Discussion

In this large sample of mixed-ancestry South Africans from Cape Town, selected miRNAs including miR-1299, miR-30e-3p, miR-126-3p significantly correlated with each other, and with 2-h post-OGTT glucose, but not with fasting glucose, HbA1c, fasting and 2-h insulin, after accounting for the effect of age, gender and BMI. In multivariable logistic regressions, the three miRNAs were consistently and continuously associated with prediabetes, while only miR-126-3p showed an association with T2DM. ROC analyses confirmed that the three miRNAs had a significant overall predictive ability in diagnosing prediabetes, diabetes and the combination of both (dysglycaemia), with AUC being always significantly higher for miR-126-3p across the three biomarkers, and always higher for prediabetes diagnosis across the three outcomes. Compared with HbA1c, miR-126-3p exhibited a better diagnostic ability in identifying prediabetes, while the two markers performed equally for dysglycaemia diagnosis, and HbA1c outperformed miR-126-3p for diabetes diagnosis. Altogether, our findings, if confirmed, would tend to suggest that miR-126-3p has a potential to play a role in diabetes risk screening strategies, particularly in the detection of people with non-diabetic range dysglycaemia, and for whom lifestyle interventions can prevent the progression to the full stage of the disease.

Human studies have suggested that altered miRNA patterns probably precede or appear at the early stages of diabetes [6,15,16]. For example, in the Bruneck study cohort, miR-126 was found to be reduced in the plasma of prevalent diabetes mellitus patients and this decrease of miR-126 preceded the manifestation of diabetes [6]. miR-126 is an endothelial cell-restricted miRNA which mediates vascular development and angiogenesis [17,18]. Apart from its role in governing vascular integrity and wound repair [19,20], miR-126 has also been reported to be associated with diabetes, specifically, micro/macrovascular complications [6,20,21,22,23,24,25]. As a result, miR-126 has been touted as a promising marker which holds the potential for diagnosis and therapeutic management of hyperglycaemia [22,23,24,25]. In our study, miR-126 was significantly increased in both DM and prediabetes compared to normotolerant individuals, and significantly correlated positively with 2-h post-OGTT. These findings are similar to our previous genome-wide miRNA profiling in which miR-126-3p was 1.74- and 1.53-fold upregulated in IGT compared to normotolerant and T2DM patients, respectively [9]. In contrast, lower circulating levels of miR-126 in prediabetes or diabetes versus normotolerant subjects have been reported [6,7,8]. The major difference between these studies and ours is that we extracted miRNA from whole blood, while others have used plasma or serum. Whole blood contains RNA from red blood cells, platelets and peripheral blood mononuclear cells (PBMCs). It has been demonstrated that the sample type affects the miRNA profile and/or expression levels [26,27]. For example, the release of miRNAs from platelets and blood cells was thought to result in an increased concentration of miRNAs in serum compared to plasma [28]. Despite these differences, our data is in agreement that miR-126 is a potential biomarker for prediabetes and our data show that its performance is superior to that of glycated haemoglobin in identifying individuals with prediabetes. HbA1c and fasting plasma glucose levels are commonly recognized as screening and diagnostic indices for diabetes and glucose intolerance. Although fasting glucose still remains a superior and standardised method, its use alone is not sufficient for the detection of all prediabetes individuals unless an OGTT is performed. Therefore, if our findings are proven and validated by others, the use of miR-126 might be of significant value in identifying these subjects in whom interventions that can prevent progression to overt diabetes can be initiated.

The other two miRNAs, miR-1299 and miR-30e-3p, were also associated with prediabetes, however their diagnostic performance was inferior to that of miR-126-3p. miR-1299 has been shown to be linked with cancers such as prostate cancer, as well as hepatocellular carcinoma. Both are thought to be involved in tumour suppression, and significantly reduced expressions have been observed in cancer cells from these patients compared to controls [29,30]. miR-1299 has also been seen to play a regulatory role in rheumatic heart disease (RHD), with significant upregulation in the RHD patients compared to healthy controls [31]. With respect to DM, there is limited information regarding miR-1299. In one study, the mononuclear cell miRNA profiles of type 1 and type 2 diabetes patients compared to healthy controls were evaluated. According to the target prediction and functional analysis, miR-1299 exhibited pathways related to T2DM, as well as pathways associated with diabetic complications (RET-HIF-1 signalling pathway) [32]. In our study, we observed a significant upregulation of miR-1299 in prediabetics versus NGT individuals. The miR-30e family has been reported to play a vital role in inhibiting cell proliferation, and studies have linked downregulation of miR-30e with various cancers [33,34]. It has also been shown to regulate renal function [1]. In their study, Wang and co-workers investigated the expression and clinical significance of plasma miRNAs in the pathogenesis and progression of diabetic nephropathy. They observed significantly low miR-30e plasma levels in diabetic patients at early stages of diabetic nephropathy versus healthy controls [1]. Hence, our findings of marked expression of miR-30e 3p in individuals with prediabetes warrants future studies that can broaden our understanding of the functional role of the miR-30e family in dysglycaemia.

The strength of our study lies in the large number of participants selected from a community-based cohort. A limitation of our study includes the absence of participants from a clinical setting for additional validation of our findings. Additionally, the OGTT was not repeated as recommended [12]. A further limitation is the disproportionality in numbers between subgroups, more notably higher numbers of the normotolerant control group, and furthermore, the sample was skewed towards female participants, all of which are common observations in community-based studies in our setting. Importantly, the results of this study must be interpreted with caution, since the endogenous control normalisation may have biased the results. Lastly, data presented here were not corrected for multiple testing, thus our findings should be interpreted with caution. In conclusion, our study has revealed an association between miR-1299, -126-3p and -30e-3p and prediabetes and the ability of miR-126-3p to significantly discriminate prediabetes beyond the performance of HbA1c. This finding deserves validation in other populations and settings, to confirm if measuring miR-126-3p could play a role in diabetes risk screening strategies.

## Figures and Tables

**Table 1:** Characteristics of the study participants.

Variable	NGT, *n* = 972	Prediabetes, *n* = 207	DM, *n* = 94	*p*-Value
Age (years)	45.2 ± 15.3	55.1 ± 13	58.4 ± 10.6	<0.001
Male, *n* (%)	284 (29.3)	42 (20.3)	19 (20.2)	
Body mass index (kg/m^2^)	27.4 ± 7.9	31.2 ± 8.7	31.3 ± 8	<0.001
Waist circumference (cm)	88.1 ± 16.8	97 ± 15.8	99.9 ± 15.5	<0.001
Hip circumference (cm)	101.1 ± 16.7	107.7 ± 16.5	107.8 ± 15.3	<0.001
Waist to hip ratio	0.9 ± 0.1	0.9 ± 0.1	0.9 ± 0.1	<0.001
Systolic blood pressure (mmHg)	131 ± 25	145 ± 27.3	145.2 ± 25.5	<0.001
Diastolic blood pressure (mmHg)	83.6 ± 15.1	89.9 ± 15.4	89.6 ± 13.6	<0.001
Fasting glucose (mmol/L	4.7 ± 0.5	5.4 ± 0.7	8.3 ± 3.9	<0.001
Post 2 h glucose (mmol/L) *	5.4 (4.5; 6.3)	8.6 (8; 9.6)	12.9 (11.6; 16.8)	<0.001
HbA1c (%)	5.6 ± 0.5	5.9 ± 0.5	7.4 ± 2.1	<0.001
HbA1c (mmol/mol)	37.7	41.0	57.4	
Fasting insulin (mIU/L)	7.6 ± 7.2	11.3 ± 11.3	14.4 ± 29.5	<0.001
Post 2-h insulin (mIU/L) *	30.5 (16; 53.6)	71.6 (42.3; 113.2)	50.5 (29.1; 79.4)	<0.001
Triglycerides (mmol/L) *	1.1 (0.8; 1.5)	1.4 (1; 1.8)	1.4 (1.1; 2.4)	<0.001
HDL-cholesterol (mmol/L)	1.4 ± 0.4	1.4 ± 0.4	1.3 ± 0.5	0.640
LDL-cholesterol (mmol/L)	3.1 ± 1	3.3 ± 0.9	3.5 ± 1.1	<0.001
usCRP (mg/L)	3.4 (1.3; 7.7)	5 (2.2; 11.0)	6.5 (3.3; 13.1)	<0.001
Cotinine (ng/mL) *	120.5 (10; 285.3)	10 (10; 271.5)	10 (10; 183)	<0.001
GGT (IU/L)	27 (19; 42)	31 (22; 53)	42 (25.5; 76)	<0.001
Current smokers, *n*(%)	540 (57.8)	99 (48.3)	29 (32.6)	<0.001
Current drinker, *n*(%)	322 (33.3)	56 (27.5)	15 (16.1)	0.002

* Median (25th, 75th percentile); NGT, normal glucose tolerance; HDL-cholesterol, high-density lipoprotein cholesterol; LDL-cholesterol, low-density lipoprotein cholesterol; usCRP, ultra-sensitive *C*-reactive protein; GGT, γ-Glutamyltransferase.

**Table 2:** Fold change analysis, 2^−ΔΔCt^ between the glucose tolerance groups.

MicroRNA	Prediabetes vs. NGT	DM vs. NGT	DM vs. Prediabetes
**RT-qPCR**			
miR-1299	4.17 ± 0.10	1.99 ± 0.13	0.48 ± 0.06
miR-30e-3p	3.22 ± 0.07	1.32 ± 0.17	0.41 ± 0.13
miR-126-3p	3.12 ± 0.11	1.75 ± 0.03	0.56 ± 0.09
**NGS Fold Changes** [9] *			
miR-1299	5.38 ± 0.23	1 ± 0.90	0.72 ± 0.88
miR-30e-3p	2.40 ± 0.03	1.78 ± 0.1	0.51 ± 1.15
miR-126-3p	1.74 ± 0.10	1 ± 1.2	1.53 ± 0.05

NGT, normal glucose tolerance; DM, diabetes mellitus; NGS, next generation sequencing. * Prediabetes only included individuals with impaired glucose tolerance.

**Table 3:** Partial Spearman’s correlation coefficients adjusted for age, sex and body mass index (BMI).

Variable	miR-1299		miR-30e-3p		miR-126-3p	
	*r*	*p*-Value	*r*	*p*-Value	*r*	*p*-Value
miR-1299	1.000		0.712	0.000	0.731	<0.001
miR-30e-3p	0.712	<0.001	1.000		0.965	<0.001
miR-126-3p	0.731	<0.001	0.965	<0.001	1.000	
Waist circumference (cm)	−0.465	0.039	−0.471	0.036	−0.444	0.050
Hip circumference (cm)	0.150	0.527	0.027	0.909	0.053	0.823
Waist hip ratio	−0.113	0.635	−0.076	0.751	−0.083	0.727
Systolic blood pressure (mmHg)	0.198	0.403	0.197	0.406	0.193	0.415
Diastolic blood pressure (mmHg)	0.201	0.395	0.171	0.472	0.161	0.497
Fasting glucose (mmol/L)	0.176	0.457	0.264	0.261	0.253	0.281
Post 2-h glucose (mmol/L)	0.369	0.109	0.399	0.082	0.429	0.059
HbA1c (mmol/mol)	0.072	0.764	0.055	0.819	0.061	0.799
Fasting insulin (mIU/L)	0.265	0.258	0.292	0.211	0.307	0.187
Post 2-h insulin (mIU/L)	0.202	0.392	0.214	0.364	0.241	0.306
Triglycerides-S (mmol/L)	−0.049	0.839	0.015	0.949	0.071	0.765
HDL-cholesterol (mmol/L)	0.458	0.042	0.452	0.045	0.401	0.079
LDL-cholesterol (mmol/L)	0.070	0.771	0.031	0.895	0.047	0.845
usCRP (mg/L)	0.185	0.435	0.199	0.400	0.165	0.488
Cotinine (ng/mL)	0.434	0.056	0.393	0.086	0.381	0.098
GGT (IU/L)	0.117	0.623	0.125	0.598	0.121	0.610

HDL-cholesterol, high-density lipoprotein cholesterol; LDL-cholesterol, low-density lipoprotein cholesterol; usCRP, ultra-sensitive *C*-reactive protein; GGT, γ-Glutamyltransferase.

**Table 4:** Multivariate regression analysis of miRNAs for the presence of prediabetes and diabetes.

MicroRNA	Prediabetes			DM		
	OR	95% CI	*p*-Value	OR	95% CI	*p*-Value
miR-1299 *						
Model 1	1.37	(1.20; 1.55)	<0.001	1.12	(0.89; 1.39)	0.338
Model 2	1.37	(1.20; 1.56)	<0.001	1.11	(0.89; 1.40)	0.354
Model 3	1.41	(1.22; 1.61)	<0.001	1.14	(0.91; 1.44)	0.254
Model 4	1.41	(1.21; 1.64)	<0.001	1.17	(0.92; 1.49)	0.213
Model 5	1.26	(1.05; 1.50)	0.012	0.92	(0.56; 1.51)	0.744
Model 6	1.31	(1.07; 1.60)	0.009	0.87	(0.48; 1.56)	0.634
miR-30e-3p *						
Model 1	2.10	(1.78; 2.48)	<0.001	1.19	(0.89; 1.59)	0.241
Model 2	2.17	(1.83; 2.58)	<0.001	1.26	(0.94; 1.69)	0.117
Model 3	2.16	(1.82; 2.57)	<0.001	1.26	(0.94; 1.69)	0.127
Model 4	2.11	(1.77; 2.51)	<0.001	1.20	(0.88; 1.65)	0.247
Model 5	1.94	(1.37; 2.74)	<0.001	1.29	(0.69; 2.41)	0.433
Model 6	2.04	(1.36; 3.08)	0.001	1.32	(0.68; 2.59)	0.414
miR-126-3p **						
Model 1	2.07	(1.85; 2.33)	<0.001	1.46	(1.25; 1.70)	<0.001
Model 2	2.15	(1.90; 2.43)	<0.001	1.53	(1.31; 1.80)	<0.001
Model 3	2.13	(1.88; 2.41)	<0.001	1.52	(1.29; 1.78)	<0.001
Model 4	2.05	(1.81; 2.33)	<0.001	1.43	(1.21; 1.69)	<0.001
Model 5	1.91	(1.51; 2.41)	<0.001	1.65	(1.19; 2.30)	<0.001
Model 6	2.04	(1.56; 2.68)	<0.001	1.80	(1.25; 2.60)	<0.001

Model 1: Crude; Model 2: included age and sex; Model 3: included age, sex and BMI; Model 4: included age, sex, BMI, SBP, triglycerides, HDL- and LDL-cholesterol; Model 5: included age, sex, BMI, SBP, triglycerides, HbA1c, 2 h post-glucose, cotinine, HDL- and LDL-cholesterol; Model 6: included age, sex, waist circumference, 2 h post-glucose, fasting insulin, HDL-cholesterol and cotinine * calculated for 0.01-unit increase; ** calculated for 1-unit increase.

**Table 5:** Multivariate regression analysis of miRNAs for diabetes using prediabetes as reference.

MicroRNA	Prediabetes			DM		
	OR	95% CI	*p*-Value	OR	95% CI	*p*-Value
miR-1299 *						
Model 1	-	-	-	0.82	(0.66; 1.01)	0.065
Model 2	-	-	-	0.81	(0.65; 1.01)	0.068
Model 3	-	-	-	0.81	(0.65; 1.02)	0.068
Model 4	-	-	-	0.83	(0.66; 1.04)	0.098
Model 5	-	-	-	0.73	(0.46; 1.16)	0.188
Model 6	-	-	-	0.66	(0.38; 1.15)	0.145
miR-30e-3p *						
Model 1	-	-	-	0.57	(0.42; 0.76)	<0.001
Model 2	-	-	-	0.58	(0.43; 0.78)	<0.001
Model 3	-	-	-	0.58	(0.43; 0.78)	<0.001
Model 4	-	-	-	0.57	(0.42; 0.78)	<0.001
Model 5	-	-	-	0.67	(0.40; 1.14)	0.138
Model 6	-	-	-	0.65	(0.38; 1.11)	0.117
miR-126-3p **						
Model 1	-	-	-	0.70	(0.61; 0.82)	<0.001
Model 2	-	-	-	0.71	(0.62; 0.83)	<0.001
Model 3	-	-	-	0.71	(0.61; 0.83)	<0.001
Model 4	-	-	-	0.70	(0.59; 0.81)	<0.001
Model 5	-	-	-	0.87	(0.69; 1.10)	0.235
Model 6	-	-	-	0.88	(0.69; 1.13)	0.328

Model 1: Crude; Model 2: included age and sex; Model 3: included age, sex and BMI; Model 4: included age, sex, BMI, SBP, triglycerides, HDL- and LDL-cholesterol; Model 5: included age, sex, BMI, SBP, triglycerides, HbA1c, 2 h post-glucose, cotinine, HDL- and LDL-cholesterol; Model 6: included age, sex, waist circumference, 2 h post-glucose, fasting insulin, HDL-cholesterol and cotinine * calculated for 0.01-unit increase; ** calculated for 1-unit increase.

**Table 6:** Performance of miR-126-3p and HbA1c in predicting dysglycaemia.

Performance Measure	Dysglycemia		Prediabetes		Diabetes	
	miR-126-3p	HbA1c	miR-126-3p	HbA1c	miR-126-3p	HbA1c
AUC	0.743 (0.705–0.781)	0.753 (0.717–0.788)	0.784 (0.742–0.827)	0.695 (0.652–0.739)	0.646 (0.576–0.717)	0.861 (0.812–0.909)
Threshold	1.41 (1.25–1.84)	5.95 (5.75–6.05)	1.78 (1.38–3.07)	5.75 (5.75–9.95)	1.31 (0.60–1.42)	6.05 (6.05–6.45)
Sensitivity	0.628 (0.570–0.683)	0.591 (0.497–0.733)	0.616 (0.545–0.683)	0.598 (0.461–0.721)	0.556 (0.447–0.660)	0.761 (0.611–0.859)
Specificity	0.737 (0.708–0.765)	0.824 (0.670–0.893)	0.804 (0.777–0.828)	0.707 (0.643–0.846)	0.708 (0.678–0.737)	0.837 (0.807–0.959)
PPV	0.424 (0.391–0.458)	0.519 (0.410–0.616)	0.401 (0.361–0.442)	0.335 (0.234–0.430)	0.152 (0.127–0.182)	0.284 (0.239–0.566)
NPV	0.866 (0.847–0.882)	0.865 (0.846–0.891)	0.907 (0.892–0.921)	0.894 (0.875–0.916)	0.944 (0.930–0.955)	0.978 (0.968–0.986)
Accuracy	0.718 (0.686–0.737)	0.771 (0.685–0.810)	0.760 (0.734–0.784)	0.702 (0.647–0.789)	0.684 (0.655–0.711)	0.833 (0.805–0.938)
*p*-value for AUC Comparison	0.281		0.048		<0.001	

AUC, area under the receiver operating characteristic curve; PPV, positive predictive value; NPV, negative predictive value.

## Data Availability

The datasets generated and/or analysed during the current study are not publicly available due to the terms of consent to which participants agreed but are available from the principal investigator (T.E.M.) of the main study on reasonable request.
